# HABS-BLOCKS©, a Floating, Slow-Release Glucose Source, Promoted the Growth of Heterotrophic Bacteria Relative to Toxic Cyanobacteria in Lake Water Mesocosms

**DOI:** 10.4236/jwarp.2024.1612044

**Published:** 2024-12-23

**Authors:** Stephen Vesper, David Linz, Ian Struewing, Jingrang Lu

**Affiliations:** United States Environmental Protection Agency, Center for Environmental Measurement and Modeling, Cincinnati, USA

**Keywords:** Glucose, Cyanobacteria, 16S Amplicon Sequencing, Microbial Community, Heterotrophs

## Abstract

Previously, we demonstrated that the addition of glucose to lake water could alter the composition of the microbial community so that heterotrophic bacteria came to dominate the cyanobacteria. To target the glucose additions to the euphotic zone, a floating, slow-release glucose source, designated HABS-BLOCKS©, was created. HABS-BLOCKS© consist of blocks of pumice stone, vacuum infiltrated with glucose, and covered in layers of soy wax. In this study, the HABS-BLOCKS© were tested in 7-liter mesocosm vessels (n = 4) that received an initial 750 ml of lake water, followed by weekly additions of 500 ml of freshly collected lake water. Three HABS-BLOCKS© were added to each of two replicate mesocosms. For controls, one mesocosm was left untreated and one mesocosm received three “Dummy” HABS-BLOCKS© (contain no glucose). During a ten-week experiment, 25 ml samples were obtained from each mesocosm weekly, which were then filtered, frozen and latter processed for 16S rRNA sequencing. *Planktothrix* and *Cyanobium* were the most abundant cyanobacteria in the lake water. Within three weeks of the start of the experiment, the bacterial community in the HABS-BLOCKS© treated mesocosms became dominated by heterotrophic bacteria, e.g., *Asticcacaulis*, relative to the control mesocosms. Heterotrophic domination in the HABS-BLOCKS© treated mesocosms continued for the rest of the experiment. HABS-BLOCKS© appears to provide a competitive edge for the heterotrophic bacteria that allows them to dominate the toxin-producing cyanobacteria.

## 1. Introduction

Harmful cyanobacterial blooms (HCBs), also called harmful algal bloom (HABs), threaten aquatic ecosystems [1]. These blooms are reportedly increasing in size and frequency worldwide [2]. The addition of nitrogen and phosphorous compounds to water bodies and a warming climate are believed to be contributing to this increase [3] [4]. As steps are taken to reduce nitrogen and phosphorous pollution and to mitigate climate change, there is an immediate need to reduce the environmental and health impacts of toxic cyanobacteria.

Hydrogen peroxide is widely used to control algal blooms by killing the cyanobacteria, but hydrogen peroxide treatment also impacts other forms of aquatic life. Piel *et al*. [5] used hydrogen peroxide to control cyanobacteria in three lakes in the Netherlands. Although the treatment nearly eliminated the cyanobacteria, new blooms developed in each of the lakes within several weeks and required repeated treatments. As collateral impacts, rotifer populations strongly declined, cladocerans were mildly affected while copepods were least impacted. In addition to being toxic, hydrogen peroxide has other limitations. Chen *et al*. [6] used hydrogen peroxide to control cyanobacteria in a 7-day mesocosm experiment and found that the dominant Cyanobacterium changed from *Dactylococcopsis*, a non-toxic genus, to the toxic *Oscillatoria* genus. Lusty and Gobler [7] also found that hydrogen peroxide reduced but did not fully eliminate the cyanobacteria. Therefore, less toxic and longer lasting bloom treatments are needed.

Previously we showed that glucose addition to lake water promoted the relative abundance of heterotrophic bacteria compared to cyanobacteria in a two-week experiment in small vessels (200 ml) [8]. Similar results were obtained in a 10-week experiment using larger mesocosm vessels (7-liter) [9]. Unlike HCB/HAB treatment with hydrogen peroxide, treatment of lake water with glucose does not kill the cyanobacteria. Rather, glucose treatment appears to allow heterotrophic bacteria to out-compete the cyanobacteria [9].

Adding large quantities of glucose to a lake is not practical and might cause problems like eutrophication. To target the glucose additions to the eutrophic zone for extended treatments, a method of glucose delivery was needed. In this study, we report on the development of a floating, slow-release glucose agent, designated HABS-BLOCKS©, which were designed to supply glucose to the euphotic zone for extended periods.

Studying bacterial communities in mesocosms for extended periods is difficult because the transfer of water from a lake to a mesocosm vessel alters the microbial community’s dynamics. Also, the physical environment of a laboratory growth chamber cannot duplicate the dynamic conditions of a lake. To reduce the impacts of these problem and still test HABS-BLOCKS© for an extended treatment period, freshly collected lake water was added each week to the mesocosms to maintain the bacterial community members found in the lake.

Our goal in treating lake water in mesocosms with HABS-BLOCKS© was to determine if they could shift the relative abundance of the bacterial community away from toxin-producing cyanobacteria to heterotrophic bacteria, as we had observed with glucose alone. Our hypothesis is that a persistent source of glucose, like HABS-BLOCKS©, could suppress the cyanobacteria for an extended period.

## 2. Materials and Methods

### 2.1. Preparation of Slow-Release Glucose Blocks or HABS-BLOCKS©

**Figure 1.**
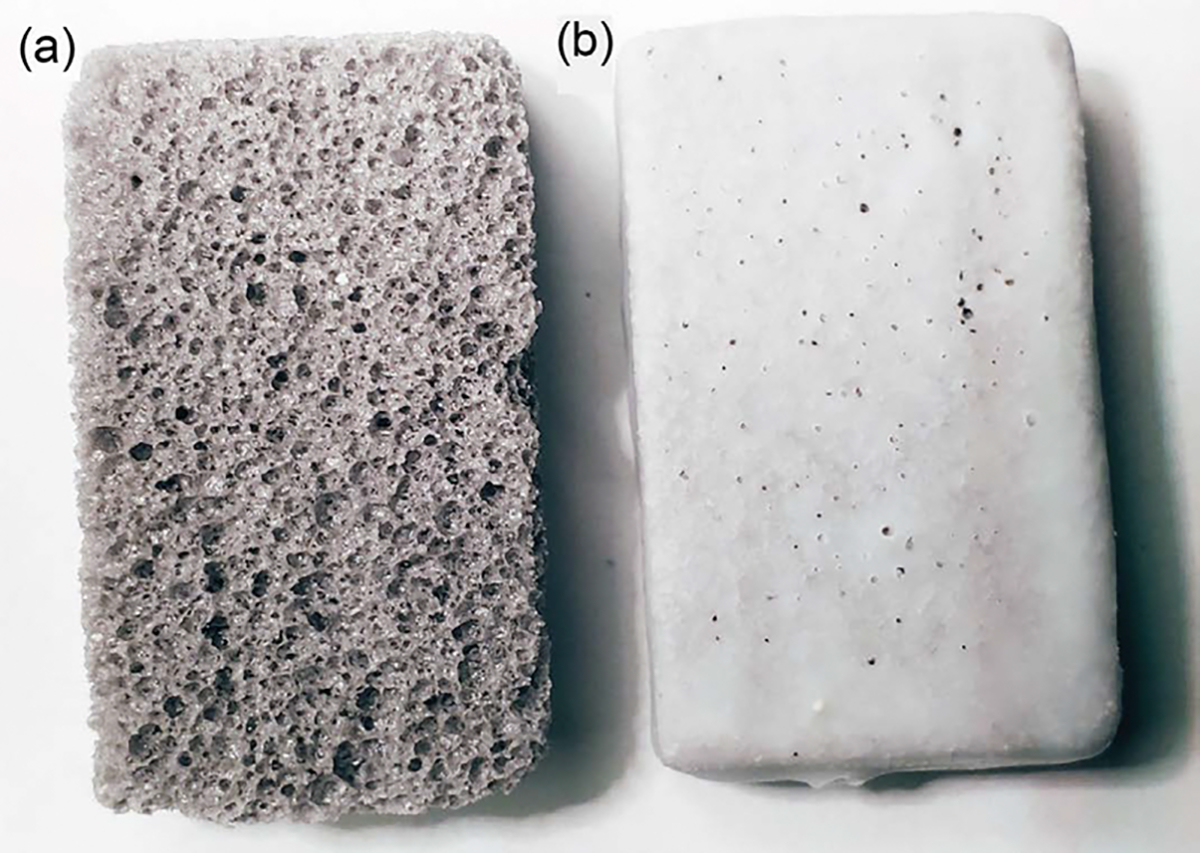
Native appearance of pumice block (a) and then following HABS-BLOCKS© creation with two layers of soy wax (b).

Pumice sticks (US Pumice Company, Chatsworth, CA) were cut into 1 × 2 × 3 cm blocks (Figure 1(a)). To infuse the glucose, the blocks were added to a saturated glucose [D-(+) Glucose SigmaUltra, Sigma-Aldrich, St. Louis, MO] solution (approximately 7 M) in a vacuum vessel. The vessel was then autoclaved at 121°C for 20 min. Immediately afterwards, a vacuum (550 Torr) was pulled on the vacuum vessel until bubbling (caused by the removal of air from the pumice blocks) stopped (~3 min). The blocks were then placed in a drying oven at 40°C to remove any residual water (~2 to 3 days). This study was conducted in 2024.

The blocks, now loaded with the glucose, were covered with soy wax (Northern Lights Natural Soy Wax, Wellsville, NY) to make them a slow-release product. The wax was melted and then each block was covered with the melted soy wax by dipping the block into the melted wax, which was then allowed to solidify. After this first layer of wax had cooled, an additional layer of wax was added by repeating the coating process to produce the HABS-BLOCKS© utilized in the study (Figure 1(b)). Each pumice block initially weighed about 2 g. HABS-BLOCKS© created for this study each contained about 3 to 4 g of glucose based on dried weight. A single layer of wax covering this size block added about 0.7 to 0.8 g to its weight and adds about 1 mm in thickness.

### 2.2. The Kinetics of Glucose Release from HABS-BLOCKS©

To measure the kinetics of the release of glucose from the HABS-BLOCKS©, 100 ml of sterile deionized was added to each of two, 250 ml beakers covered with aluminum foil. Then two HABS-BLOCKS© or two “Dummy” HABS-BLOCKS© (same wax coated pumice blocks but without glucose) were added to the beakers and incubated at 20°C on a rotary shaker at 50 RPMs. The concentration of glucose in each beaker was measured after 6 hr. on day one and then daily using Glucose Test Strips (Precision Laboratories, Inc., Cottonwood, AZ), following the manufacturer’s instructions. After each daily measurement of glucose concentration, the HABs-BLOCKS© or “Dummy” HABs-BLOCKS© were aseptically removed and the beaker and HABs-BLOCKS© or “Dummy” HABs-BLOCKS© were rinsed in sterile, deionized water. Then the HABS-BLOCKS© or “Dummy” HABS-BLOCKS© and another 100 ml aliquot of sterile deionized water were added to the two beakers, which were again placed on the rotary shaker for further incubation until the glucose was no longer measurable.

### 2.3. Mesocosm Study with HABs-BLOCKS©

Water from the William H. Harsha Lake (hereafter, Lake), previously described in detail [10], was collected on June 29, 2023, from near the Lake’s surface (~0.5 m depth), using plastic water jugs, which were pre-rinsed using 5% hydrochloric acid and deionized water, as previously described [11]. Then 750 ml of the Lake water were added to each of four 7-liter polypropylene vessels (8SFSPP, CAM-BRO, Huntington Beach, CA). Each mesocosm vessel was then covered with a transparent xerography sheet (Skillcraft, Greensboro, NC). The mesocosms were placed in an environmental chamber (Percival/166LLVL) with the following growth conditions: the light intensity was 44.02 μmol photons/m^2^/s (measured using a LICOR LI-1500) with a 16/8-hour light/dark cycle at a constant temperature of 25°C and ambient air-exchange.

After the initial 750 ml of Lake water were added to each mesocosm, the water was allowed to equilibrate for 24 hr. Then a time-zero, 25 ml sample was collected from each mesocosm. These 25 ml samples were filtered through Durapore polyvinylidene fluoride (PVDF) filters, with 0.45 μm pore size (MilliPore, Foster City, CA). Each filter was inserted into a 2-ml Matrix Lysis-A tube (MP Biomedicals, LLC, Santa Ana, CA) and stored at −80°C until analyzed.

For the experiment, the mesocosms were mixed daily for 30 min using stir plates. Control mesocosm 1 was left untreated and Control mesocosm 2 received three “Dummy HABS-BLOCKS©”. Mesocosms 3 and 4 were treated with three HABS-BLOCKS© each. Immediately after the 30 min daily mixing period, weekly 25 ml aliquots were collected from each mesocosm. Just as was done for the time-zero aliquot, these aliquots were filtered and stored at −80°C until analyzed. After each weekly sampling event, each mesocosm received 500 ml of freshly collected Lake water. By the end of the experiment, each mesocosm contained approximately 5.5 liters of Lake water.

### 2.4. Processing Filters for 16S rRNA Analysis

The tubes containing the filters were retrieved from the −80°C freezer and the cells lysed using a Mini-Beadbeater-16 (BioSpec Products, Inc., Bartlesville, OK, USA) by milling for one min at 5000 RPMs; then the tubes were placed in an ice-bucket for 5 min. Afterwards, the cap on the micro-tube was loosened to allow for air equilibration and a hole was made in the bottom of the micro-tube using a sterile syringe needle (22 gauge). The tubes were placed in pre-drilled holes in the tops of sterile 15 ml centrifuge tubes (Corning CentriStar, Corning Inc., Corning, NY). This assembly was centrifuged at 2000 RPMs for 2 min resulting in approximately 600 μl of DNA extract to be transferred to the bottom of the 10 ml tube. The extract was retrieved from the bottom of the 10 ml tube and placed in a 1.6 ml microfuge tube and then centrifuged at 14,000 RPMs for 3 min and then 500 μl of supernatant transferred to the filter unit of the Qiagen Allprep DNA/RNA Mini Kit (Qiagen) for DNA purification, following the manufacturer’s instructions.

### 2.5. Amplicon Sequencing

Library preparation and sequencing were performed to target the 16S rRNA v3–4 region. The methods were described by Bagley *et al*. [12] with the following modifications. The first round PCR were performed with 22 μL Accuprime pfx supermix (Thermo-Fisher, Waltham, MA), 0.5 μL of each primer, and 2 μL of DNA. After gel confirmation of amplification products, PCR products were cleaned with 19 μL of AMPure XP beads (Beckman Coulter, Brea, CA) added to 23 μL of the PCR products and eluted in 35 μL of 10 mM Tris pH 8.5. PCR products were normalized to 5 ng/μL. An index PCR was performed using 22 μL Accuprime pfx supermix, 0.5 μL of 10 μM of each index primer, and the product cleaned using 27.5 μL AMPure XP added to 25 μL of PCR product and eluted with 35 μL of 10 mM Tris pH 8.5. Samples were normalized to a concentration of 4 nM and 5 μL of each were combined to make the final library. The pooled library was sequenced using a 600 cycle V3 MiSeq sequencing kit (# MS-102–3003, Illumina, San Diego, CA, USA) according to manufacturer’s protocol, using 2 × 300 paired-end sequencing.

### 2.6. Amplicon Processing

Raw demultiplexed reads, with adapters removed, were then processed using the software suite QIIME 2 2021.4.0 [13] and the raw sequence data quality filtered, chimeras removed, and denoised with DADA2 (via q2-dada2) [14]. Taxonomy was assigned to amplicon sequence variants (ASVs) based on the Silva 138 SSU reference using the q2-feature-classifier [15] and classify-sklearn naïve Bayes taxonomy classifier [16] [17]. Qiime2 artifacts were then moved to R v4.1.2 using the qiime2R package for further analysis [18].

### 2.7. Sequence Data Analysis

Analysis of the final sequence dataset was performed in R v4.1.2 [19] using the packages phyloseq (v1.38.0) [20], vegan (v2.5.7) [21], and ggplot2 (v3.3.5) [22]. Samples were initially pruned of non-bacterial or unidentified taxa. Replicates for each sample were initially examined and, after determining consistency among replicates, merged for subsequent analysis. For certain components of our analysis, low abundance taxa (<5%) were removed from the dataset.

## 3. Results

**Figure 2.**
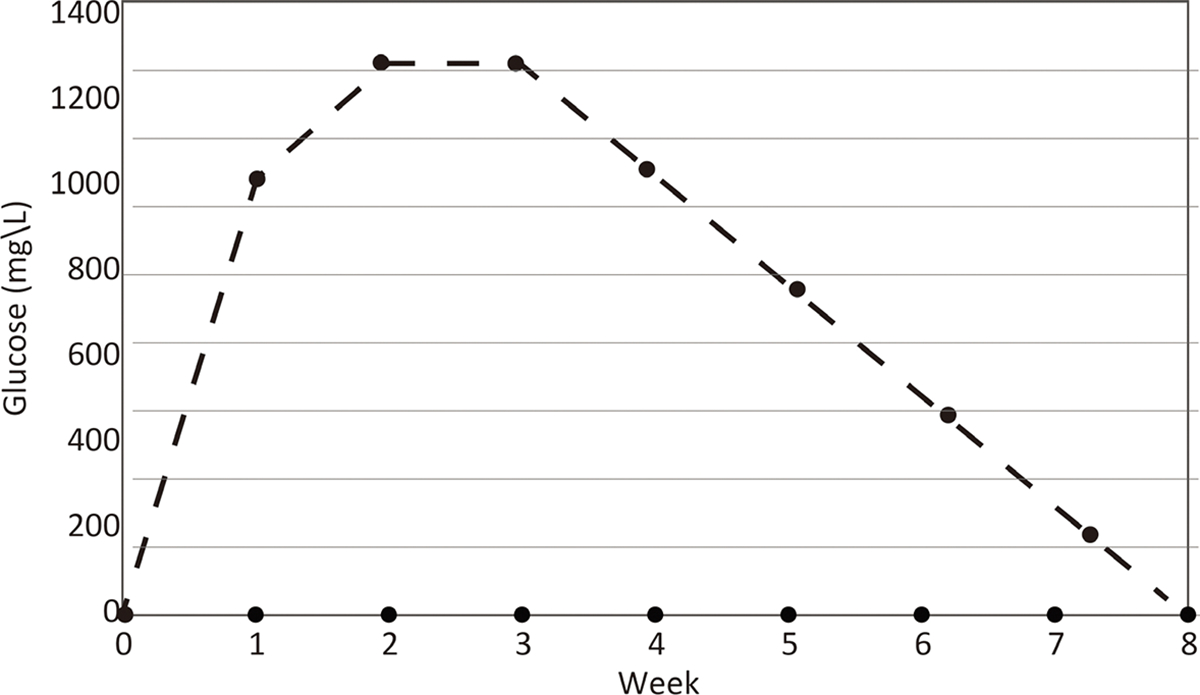
Log of the glucose concentration in daily refreshed 100 ml sterile water treated with two HABS-BLOCKS©.

HABS-BLOCKS© with two layers of soy wax were found to release an average of about 100 mg glucose/100ml of sterile water for 28 days (Figure 2). Glucose was never detected in the “Dummy” HABS-BLOCKS© treated water.

The addition of HABS-BLOCKS© to mesocosms containing Lake water altered the bacterial community structure. For example, across sampling dates, bacterial communities in the HABS-BLOCKS© treated mesocosms 3 and 4 and Control mesocosms 1 and 2 were significantly different in their composition (Bray-Curtis, ANOSIM; R = 0.401, p < 0.001). When the data from all sampling dates were combined, differences were found for all pairwise comparisons between HABS-BLOCKS© treated and Control mesocosms (Table 1). However, no significant differences were observed when comparing within Control mesocosms (1 vs 2) and HABS-BLOCKS© treated mesocosms (3 vs 4) (Table 1). Therefore, for subsequent analyses, the pairs of data from the two Control mesocosms were merged and the pairs of data from the two HABS-BLOCKS© treated mesocosms were merged.

**Figure 3.**
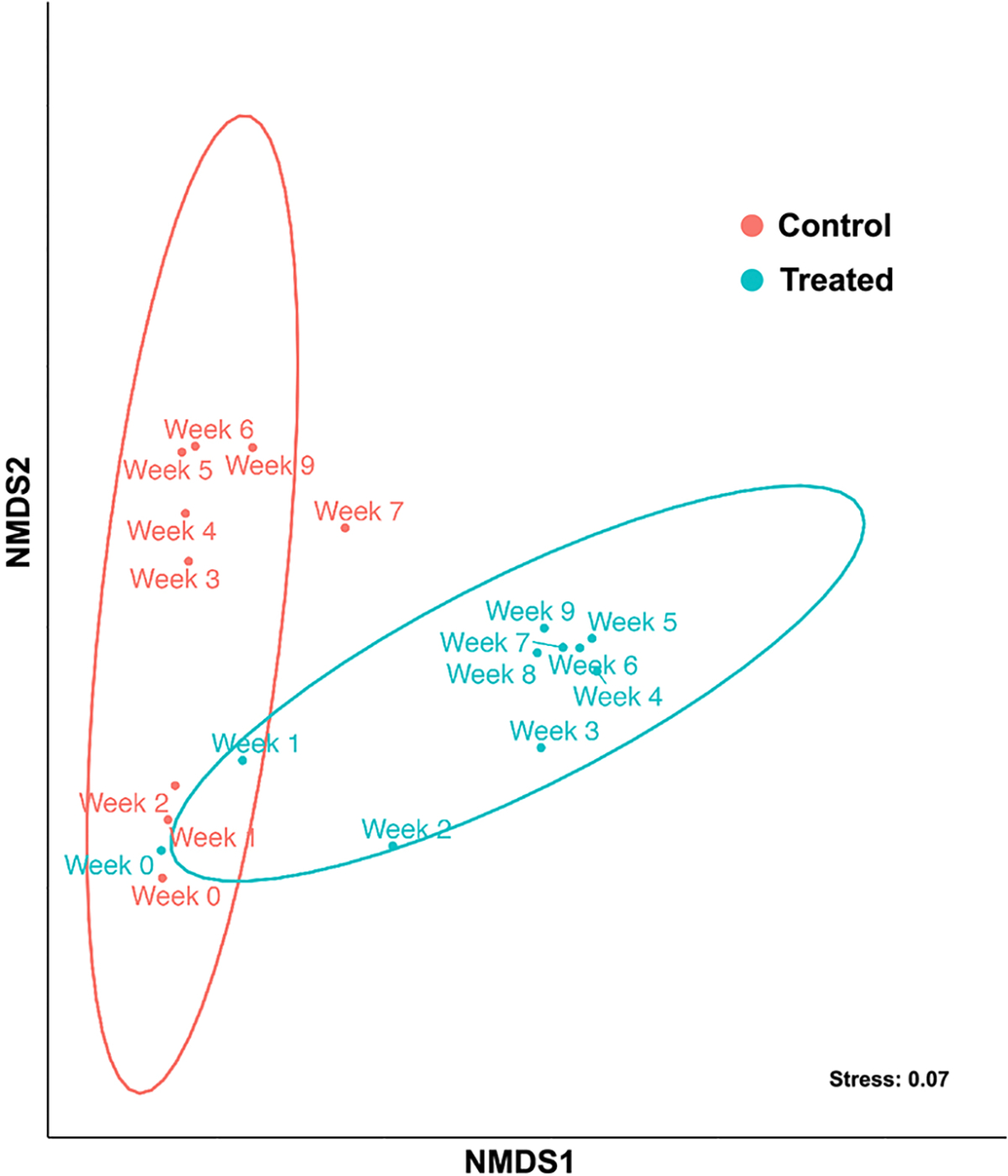
Beta diversity of microbial community composition in HABS-BLOCKS© treated and Control mesocosms across weeks shown via a non-metric multidimensional scaling (NMDS) plot. Category of treatment is indicated by color.

Next, we examined the bacterial community compositions in the HABS-BLOCKS© treated and Control mesocosms using the 16S rRNA sequence data. Ordination was performed using non-metric multidimensional scaling (NMDS) to examine the differences, *i.e.* the beta diversity, in the bacterial community at each week of the experiment. The bacterial communities in the HABS-BLOCKS© treated and Control mesocosms were found to be significantly different across sampling dates (Bray-Curtis, ANOSIM; R = 0.6145 and p = 0.002) (Figure 3).

**Figure 4.**
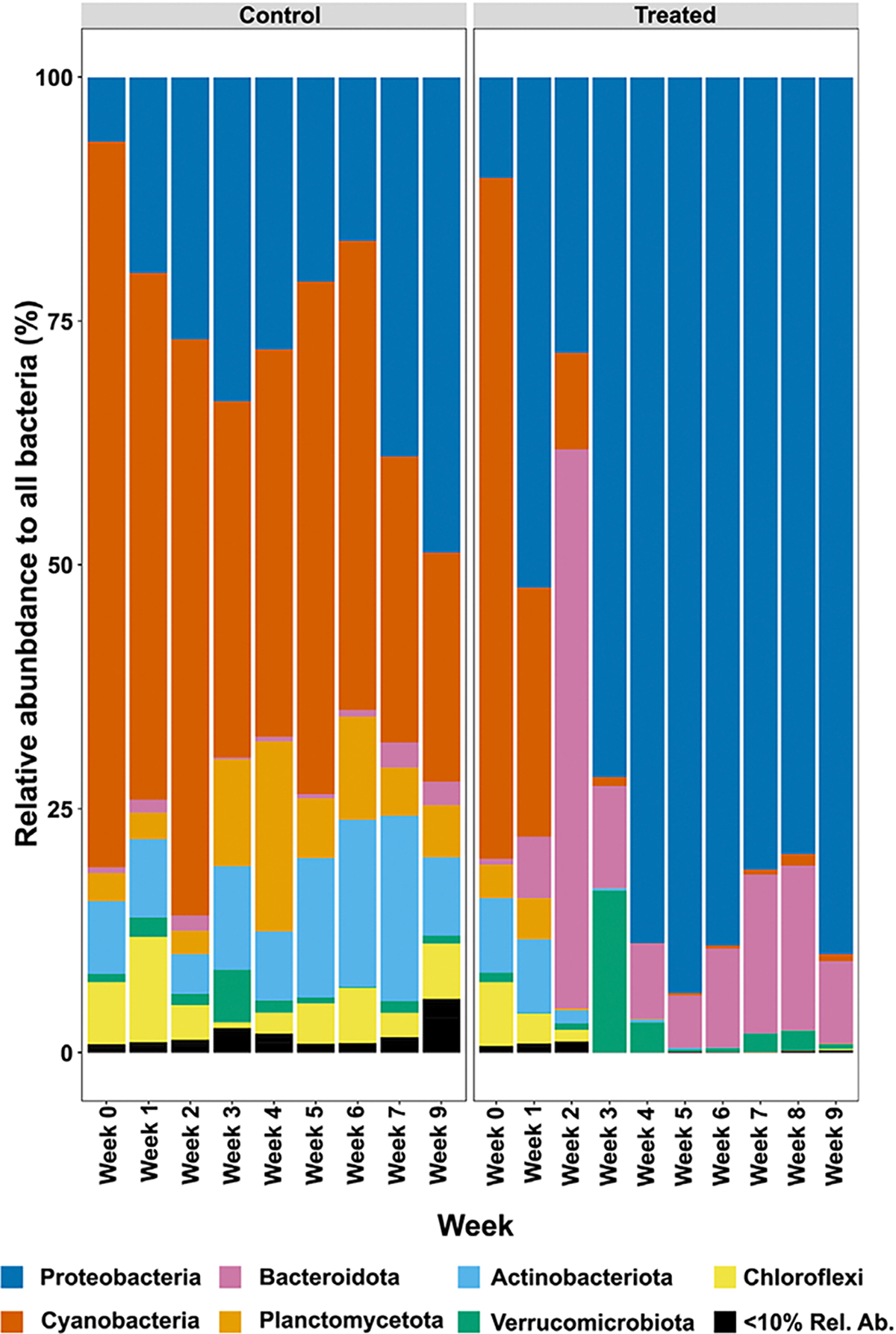
Changes in the relative abundance of bacterial phyla in Control mesocosms compared to HABS-BLOCKS© treated mesocosms.

Then, the 16S rRNA sequence data was used to compare the relative abundance of the bacterial phyla in the HABS-BLOCKS© treated and Control mesocosms at each week of the experiment (Figure 4). Initially, the HABS-BLOCKS© treated and Control mesocosms were dominated by Cyanobacteria (orange, Figure 4) and Proteobacteria (blue, Figure 4). However, after about three weeks, the HABS-BLOCKS© treated mesocosms became dominated by Proteobacteria (blue, Figure 4) and Bacteroidota (pink, Figure 4). But the Cyanobacteria persisted in the Control mesocosms until the end of the experiment (orange, Figure 4).

**Figure 5.**
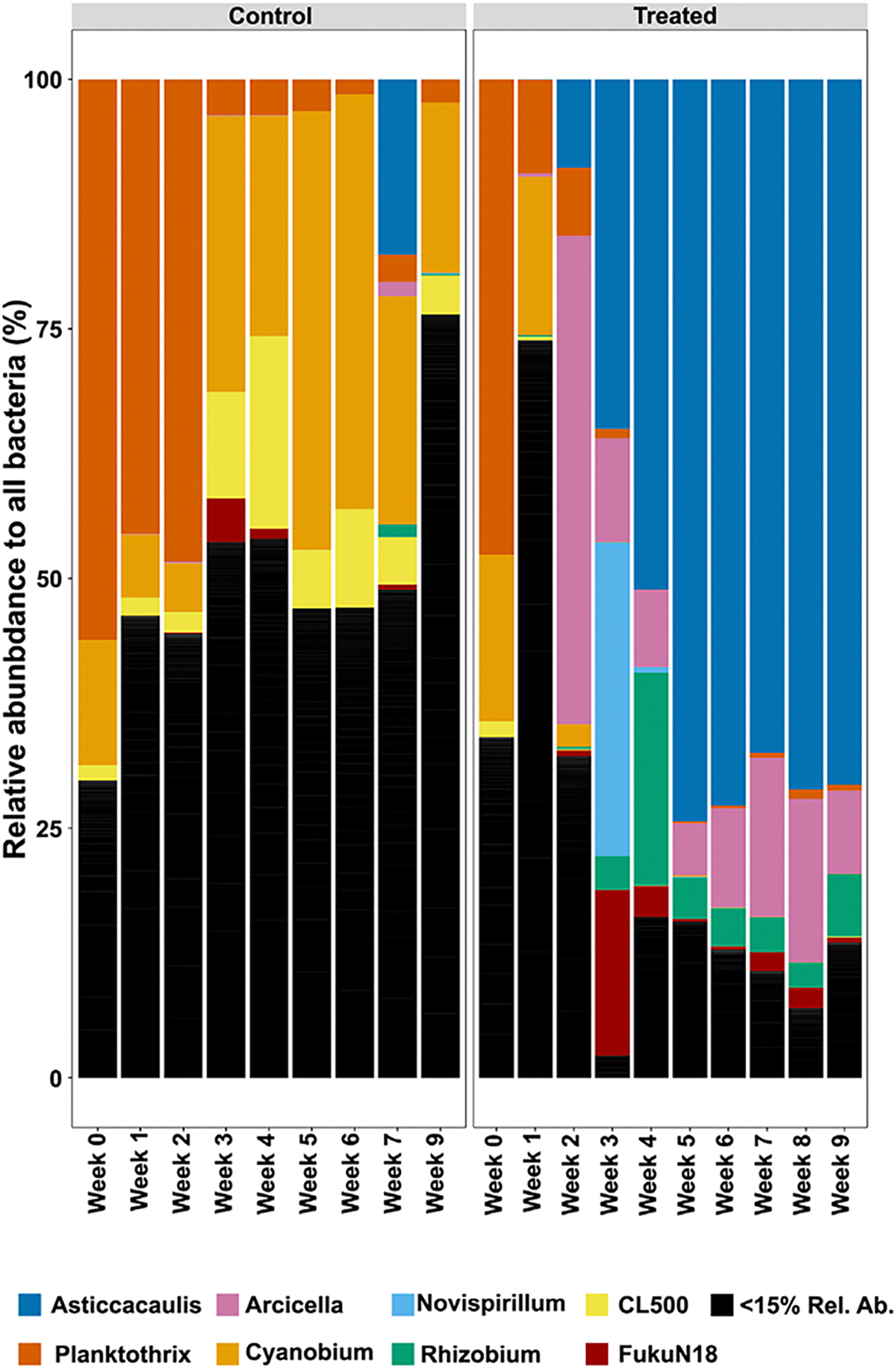
Changes in relative abundance of bacterial genera in Control mesocosms compared to HABS-BLOCKS© treated mesocosms. (NOTE: CL500–3 is s consensus genus in the phylum Planctomycetota; Fuku18 is a consensus genus in the phylum Verrucomicrobiota).

Next, the relative abundance and composition of the bacterial genera in the HABS-BLOCKS© treated and Control mesocosms were compared (Figure 5). Low abundance genera were dominant in the Control mesocosms (black bars, Figure 5). However, of the identified cyanobacteria, *Planktothrix* and *Cyanobium* were relatively abundant in the Control mesocosms for the entire experiment. Similarly, for the first two to three weeks of the experiment, the HABS-BLOCKS© treated mesocosms contained appreciable levels of *Planktothrix*, *Cyanobium*, *Arcicella*, and low abundance genera (Figure 5). But, after first two to three weeks, *Asticcacaulis* and *Arcicella* became the dominant genera in relative abundance in the HABS-BLOCKS© treated mesocosms for the remainder of the experiment (Figure 5).

## 4. Discussion

HABS-BLOCKS© are made with natural components, *i.e.*, pumice stone, glucose, and soy wax. Therefore, HABS-BLOCKS© are non-toxic and environmentally friendly since the glucose and soy wax are readily biodegradable and the pumice stones are reusable. When the glucose has been expended from the HABS-BLOCKS©, they can be collected from the water surface, cleaned in hot water to remove any remaining soy wax, and then made into more HABS-BLOCKS©.

HABS-BLOCKS© float and therefore the glucose is delivered into the euphotic zone where they promoted the populations of heterotrophic Proteobacteria and Bacteroidota relative to the toxic Cyanobacteria, as we had observed previously with glucose alone [8] [9]. *Planktothrix* and *Cyanobium* were the most abundant of the identified cyanobacterial genera in the Harsha Lake water mesocosms. The association of *Planktothrix* and *Cyanobium* has been found in other lakes. In Utah Lake, Li *et al*. [23] found that among all cyanobacteria, *Cyanobium* populations were most significantly correlated with *Planktothrix* populations. In three eutrophic lakes in northern Florida, *Planktothrix* and *Cyanobium* were the most abundant cyanobacteria [24] and in a natural reservoir in Eastern Australia, *Planktothrix* and *Cyanobium* dominated in the summer [25]. Previously, we had shown that *Planktothrix* and *Microcystis* became the dominant cyanobacteria in Harsha Lake when nitrogen became limited [26]. So, our experiment may have relevance for other lakes.

After the first 2 to 3 weeks of the experiment, *Asticcacaulis* became the dominant heterotrophic bacterium in the HABS-BLOCKS© treated mesocosms. *Asticcacaulis* sp. are known for their buoyancy, which allows them to remain within the upper layer of waters [27]. This may explain why the floating HABS-BLOCKS© promoted their population. *Asticcacaulis* sp. may also have other adaptive qualities that allow it to thrive in HABS-BLOCKS© treated water. Hoang *et al*. [28] studied the response of microbial communities to the frequency of disturbance to the ecosystem, what they call the diversity-disturbance relationship (DDR). They found that *Asticcacaulis* was more abundant in the highest DDR treatment conditions. They summarized that the DDR across different microbial systems may be due to the “nutritional resources microbial communities can access and the interactions between bacteria and their environment.” Therefore, the addition of glucose may fundamentally shift the microbial community away from cyanobacteria to heterotrophs like *Asticcacaulis*.

Since this study was primarily a proof-of-concept for using HABS-BLOCKS©, there are many limitations that will require future studies. For example, many physical, chemical, and biological parameters of the water were not quantified and any negative effects of the added glucose not assessed. Possible negative impacts of adding glucose to a lake could be a reduction in dissolved oxygen in the water and an increase in dissolved solids, which could lead to eutrophication with negative impacts on other aquatic organisms. But the major limitation of the study was that mesocosms are never a true reflection of dynamic conditions in a lake, even though fresh lake water was added weekly to the mesocosms. In open water, one would expect that the cyanobacterial population would be diluted continuously as the heterotrophic bacterial population out competed the cyanobacteria. We will test this hypothesis in the future under *in situ* lake conditions.

## 5. Conclusion

HABS-BLOCKS© in mesocosms containing Lake water promoted the increased relative abundance of heterotrophic bacteria compared to the toxic cyanobacteria. However, there is much to learn before the application of HABS-BLOCKS©. We especially need to know the optimum number of HABS-BLOCKS© needed to suppress a bloom without any negative impacts.

## Figures and Tables

**Table 1.** Pairwise analysis of similarities (ANOSIM) among mesocosms with Benjamini- Hochberg adjusted p-values. Control mesocosm 1—nothing added; Control mesocosm 2— “Dummy” HABS-BLOCKS© added; Treatment mesocosms 3 and 4—HABS-BLOCKS© added.

Mesocosm Pairs	R value	Adjusted p-value

**1 vs 2**	−0.05	0.58
**1 vs 3**	0.47	0.01
**1 vs 4**	0.49	0.01
**2 vs 3**	0.58	<0.01
**2 vs 4**	0.61	<0.01
**3 vs 4**	0.09	0.12

## Data Availability

All data will be available at the NIH-PMC website. All sequence data have been deposited in the NCBI sequence read archive at accession number: PRJNA1142472.
